# Gut microbiota dysbiosis and systemic inflammation in elderly Chinese hypertensive patients: a case-control study

**DOI:** 10.3389/fimmu.2025.1662578

**Published:** 2025-11-13

**Authors:** Yiwen Cheng, Yihua Huang, Zhangcheng Zhu, Wenwen Ding, Xia Liu, Xiaocui Xu, Yangtian Chen, Ling Wang, Longyou Zhao, Zongxin Ling, Shiwei Ye

**Affiliations:** 1 State Key Laboratory for Diagnosis and Treatment of Infectious Diseases, National Clinical Research Center for Infectious Diseases, China-Singapore Belt and Road Joint Laboratory on Infection Research and Drug Development, National Medical Center for Infectious Diseases, Collaborative Innovation Center for Diagnosis and Treatment of Infectious Diseases, The First Affiliated Hospital, Zhejiang University School of Medicine, Hangzhou, Zhejiang, China; 2 Yuhang Institute for Collaborative Innovation and Translational Research in Life Sciences and Technology, Hangzhou, Zhejiang, China; 3 Department of Nursing, Lishui Second People’s Hospital, Lishui, Zhejiang, China; 4 Department of Preventive Medicine, School of Public Health and Management, Wenzhou Medical University, Wenzhou, Zhejiang, China; 5 Department of Anesthesiology, Affiliated Hospital of Nantong University, Medical School of Nantong University, Jiangsu, China; 6 Department of Intensive Care Unit, The First Affiliated Hospital, Zhejiang University School of Medicine, Hangzhou, Zhejiang, China; 7 Department of Laboratory Medicine, Lishui Second People’s Hospital, Lishui, Zhejiang, China; 8 Department of Clinical Nutrition, Lishui Second People’s Hospital, Lishui, Zhejiang, China

**Keywords:** *Blautia*, enterotype, gut dysbiosis, hypertension, inflammation

## Abstract

Hypertension (HTN) remains the leading modifiable risk factor for global mortality and morbidity. The number of adults aged 30–79 with HTN has doubled worldwide, reaching approximately 1.3 billion, with nearly half unaware of their condition. Despite available therapies, the global control rate remains unacceptably low at around 20%, particularly in low- and middle-income countries. This substantial treatment gap contributes to a high burden of preventable cardiovascular events and strains healthcare systems globally, underscoring the urgent need for more effective, accessible, and personalized management strategies. Growing evidence suggests that gut microbiota dysbiosis plays a role in HTN pathogenesis, though its mechanistic basis remains incompletely understood. In this case-control study, we investigated the gut microbiota composition of 205 elderly Chinese individuals (153 HTN patients, 52 controls) using NovaSeq sequencing and assessed systemic inflammation using multiplex immunoassays. Enterotype analysis and receiver operating characteristic (ROC) modeling were employed to identify microbial signatures. HTN patients demonstrated significant β-diversity alterations and distinct taxonomic changes, characterized by enriched *Escherichia_Shigella*, *Prevotella_9*, and *Enterococcus*, and depletion of *Blautia* and butyrate-producing genera. The *Escherichia_Shigella*-dominated enterotype (E2) was significantly more prevalent in HTN. ROC-based biomarker analysis identified *Blautia*, *Butyricicoccus*, *Lachnoclostridium*, *Prevotella_9*, and *Enterococcus* as potential diagnostic biomarkers. HTN patients also exhibited elevated pro-inflammatory cytokines such as IL-1ra and TNF-α, indicative of chronic low-grade inflammation. Correlation analysis revealed strong associations between pathobionts (e.g., *Escherichia_Shigella*) and pro-inflammatory cytokines, and between butyrate producers (*Blautia*) and anti-inflammatory mediators. These findings underscore gut dysbiosis and systemic inflammation as key pathophysiological features in elderly hypertension and provide a foundation for developing microbiota-based diagnostic and therapeutic approaches for this population.

## Introduction

Hypertension (HTN), or high blood pressure (BP), is a major global health issue, affecting around 1.3 billion adults worldwide, with particularly high prevalence in low- and middle-income countries (1, 2). Despite the availability of antihypertensive treatments, BP control remains inadequate globally, with control rates ranging from 18% to 23%, and as low as 10% in some regions of Asia and Africa. While poor adherence to treatment is a significant factor in these low rates, it also highlights the fact that the underlying causes of elevated BP are often still unclear for many individuals with HTN (2). As a leading risk factor for cardiovascular and cerebrovascular diseases including stroke, ischemic heart disease, heart failure, and myocardial infarction, HTN also plays a major role in chronic kidney disease, cognitive decline, and is a primary cause of death and disability worldwide (3, 4). Its multifactorial pathophysiology involves genetic factors, environmental influences, lifestyle habits, and dysregulations in the vascular and renal systems. Additionally, factors such as sympathetic nervous system overactivity, endothelial dysfunction, and systemic inflammation have been linked to the onset and progression of HTN (5–7).

Recent studies have shown that the gut microbiota plays a pivotal role in the development and progression of HTN (8–10). Cross-sectional human studies have consistently shown reduced α-diversity in individuals with HTN, along with an overrepresentation of specific bacterial taxa—such as *Klebsiella*, *Parabacteroides*, *Clostridium*, and *Prevotella*—that are positively correlated with elevated BP (11–13). Conversely, certain beneficial genera, including *Roseburia*, *Akkermansia*, and *Ruminococcus*, have been associated with lower BP in some cohorts, though these findings are not consistently replicated across all populations (14–20). Complementary evidence from animal models—such as spontaneously hypertensive rats (SHRs), Dahl salt-sensitive rats, high-salt-fed mice, and angiotensin II-infused mice—further supports the association between gut microbiota disruption and HTN (21–23). Notably, germ-free mice that received fecal microbiota transplantation from human hypertensive donors developed analogous microbial alterations and exhibited significantly elevated blood pressure after ten weeks (15), underscoring a potential causal role of gut dysbiosis in HTN pathogenesis.

Mechanistically, HTN-associated gut dysbiosis is characterized by a decline in beneficial short-chain fatty acid (SCFA)-producing bacteria—such as *A. muciniphila* and *Faecalibacterium prausnitzii*, coupled with an expansion of pro-inflammatory opportunistic pathobionts (24, 25). This microbial shift leads to impaired production of protective metabolites like butyrate, resulting in weakened activation of G-protein-coupled receptors (GPR41/43), which are essential for regulating renin release, maintaining endothelial function, and exerting anti-inflammatory effects (26–28). Concurrently, dysbiosis promotes the overproduction of trimethylamine (TMA) from dietary choline and L-carnitine, which is subsequently oxidized in the liver to trimethylamine N-oxide (TMAO). TMAO contributes to elevated BP by promoting endothelial dysfunction, enhancing platelet aggregation, and accelerating atherosclerosis (28, 29).

Inflammation further plays a central role in the gut-immune-vascular axis. Dysbiosis compromises intestinal barrier integrity by downregulating tight junction proteins such as occludin, thereby increasing gut permeability (30, 31). This facilitates the translocation of bacterial endotoxins like lipopolysaccharide (LPS) into systemic circulation, where they activate the TLR4/NF-κB signaling pathway in immune cells. This cascade triggers the release of pro-inflammatory cytokines, including TNF-α, IL-1β, and IL-17, which collectively promote vascular dysfunction, renal sodium retention, and sympathetic overactivity—all key mechanisms driving hypertension (32–35). Notably, high-salt diets in mice reduce the abundance of *Lactobacillus murinus* while promoting Th17 cell differentiation, thereby exacerbating salt-sensitive HTN. Supplementation with *L. murinus* has been shown to attenuate this response by modulating Th17 activity, underscoring the microbiome’s critical role in inflammatory regulation in HTN (22, 36). The metabolic shift within the dysbiotic gut environment further aggravates disease progression, marked not only by reduced SCFA levels but also by the accumulation of harmful metabolites such as TMAO and α-lipomycin—a TRPV4 antagonist—which collectively enhance systemic inflammation and impair vascular function (37–39). Recent clinical evidence, including a randomized controlled trial showing BP reduction following fecal microbiota transplantation (FMT), further substantiates the causal role of the gut microbiome in HTN (40). These insights validate the gut-immune-vascular axis as a key pathogenic pathway in hypertension and open promising avenues for personalized, microbiota-based treatments.

Understanding how gut dysbiosis influences host immunity and BP regulation is key to uncovering new mechanisms of HTN, identifying potential biomarkers, and exploring new therapeutic targets. To explore this further, we recruited 153 elderly HTN patients and 52 age- and gender-matched healthy controls (Con) from Lishui, China (**Supplementary Figure S1**). We analyzed fecal microbiota composition using high-throughput sequencing targeting the V3-V4 hypervariable regions of the 16S rRNA gene and assessed circulating levels of 27 cytokines through bead-based multiplex immunoassays. Our study also explored the relationship between HTN-associated bacterial taxa and circulating inflammatory mediators. These findings will provide new insights into HTN and potentially pave the way for non-invasive diagnostic methods and microbiota-based therapeutic strategies.

## Methods

### Participants’ enrollment

A total of 153 HTN patients (aged 61–76 years) and 52 age- and gender-matched healthy controls with normal BP were recruited from Lishui, China, between May and August 2024. The study was approved by the ethics committee of Lishui Second People’s Hospital (reference no. 20230119-01), and all participants provided written informed consent prior to inclusion. Hypertension was diagnosed according to the following criteria: systolic blood pressure (SBP) ≥140 mmHg or diastolic blood pressure (DBP) ≥90 mmHg, confirmed either through repeated examinations (41) or the use of antihypertensive medication. BP measurements were taken with participants seated, conducted by trained nurses. Three readings were recorded at 5-minute intervals using a random-zero mercury column sphygmomanometer, and the average of these readings was considered the final measurement. Exclusion criteria for the study included: age <60 years, body mass index (BMI) >28 kg/m², acute respiratory or intestinal infections, cancer, stroke, peripheral artery disease, heart failure, diabetes, intestinal surgery, inflammatory bowel disease, diarrhea, chronic pancreatitis, elevated body temperature, high white blood cell count, and the use of antibiotics, prebiotics, probiotics, symbiotics, or immunosuppressive drugs within the past month.

### Sample collection

Sample collection and processing followed standardized protocols (42). Approximately 2g of fresh fecal samples were collected in sterile plastic cups and immediately stored at -80 °C within 15 minutes of collection for microbiome analysis. Serum samples were obtained from participants’ fasting blood in the early morning. After centrifugation (1500 × g, 10 min), the serum was divided into three equal 200 µl aliquots and immediately stored at −80 °C for subsequent analysis.

### Amplicon library preparation and NovaSeq sequencing

Bacterial genomic DNA was extracted from 300 mg of homogenized fecal samples using the QIAamp DNA Stool Mini Kit (Qiagen, Hilden, Germany, Cat. No. 51604), following the manufacturer’s instructions, with additional glass-bead beating steps on a Mini-beadbeater (FastPrep; Thermo Electron Corporation, Boston, MA, USA). The protocols of amplicon library preparation, and sequencing have been described in detail in previous studies (42–46). The amplicon library was constructed to target the hypervariable V3-V4 regions of the 16S rRNA gene using standardized protocols. Negative controls (lysis buffer and kit reagents only) were processed and sequenced to assess potential contamination. The sequencing library was prepared at Hangzhou KaiTai Bio-lab, and sequencing was performed on the NovaSeq™ 6000 system (Illumina).

### Bioinformatic analysis

Following sequencing, raw data (>100 bp) with an error rate of <1% were processed and quality-controlled using QIIME2 (Quantitative Insights Into Microbial Ecology 2, v2020.11, http://qiime.org/) with default parameters (42–49). Quality control steps included trimming adapter sequences using Cutadapt v2.4 and removing low-quality reads, sequences shorter than 100 bp, and chimeric sequences using the fastq_filter module in VSEARCH v2.13.6, which preserved unique *de novo* sequence variants. Prior to analysis, the reads from each sample were normalized to ensure uniform sampling depth. Amplicon sequence variants (ASVs) were generated through denoising and quality control, including chimera removal, using the DADA2 plugin (50). Taxonomic assignment of ASVs was performed using the SILVA database (Release 138, http://www.arb-silva.de). Bacterial α-diversity metrics, including Shannon, Simpson, observed species, ACE, and Chao1 estimators, were computed at a 97% similarity threshold. β-diversity was evaluated using Bray-Curtis, Jaccard, unweighted UniFrac, and weighted UniFrac distances in QIIME2, with visualization via principal coordinate analysis (PCoA) (51). Taxa with low abundance, defined as those appearing in fewer than 50% of samples, were excluded from subsequent analysis. Gut bacterial enterotypes were employed to identify distinct bacterial communities across samples. Microbiota composition variations were assessed using the Statistical Analysis of Metagenomic Profiles (STAMP) software package v2.1.3 (52) and LEfSe (53), focusing on taxa with an average relative abundance > 0.01% for biomarker discovery (LDA score > 3.0; p < 0.05). Correlation analysis was performed using the sparse compositional correlation (SparCC) algorithm on the complete ASV table at the genus level, with networks visualized using Cytoscape v3.6.1. SparCC was selected due to its ability to uncover potential ecological interactions, such as mutualism or competition, within microbial communities. Functional predictions based on the ASV table generated by QIIME were compared with the Kyoto Encyclopedia of Genes and Genomes (KEGG) database using PiCRUSt v1.0.0. This method used ancestral-state reconstruction to predict the functional potentials of microbial communities based on their phylogenetic composition, categorizing them into KEGG pathways at levels 1-3 (54, 55).

### Multiplex cytokine analysis

Systemic immune function was assessed in participants using the Bio-Plex Pro Human Cytokine 27-plex assay kit (M500KCAF0Y, Bio-Rad, Hercules, CA, USA), following the manufacturer’s instructions (42–46, 56). This multiplex assay, based on Luminex^®^ xMAP^®^ technology, enables the simultaneous quantification of 27 immune markers, including 16 cytokines, 6 chemokines, and 5 growth factors. Measurements were conducted on the Luminex^®^ 200™ system (Bio-Rad), with fluorescence values recorded for each analyte. Serum samples were diluted 1:4 with the sample diluent buffer, and standard curves were generated using a range of known assay standards. Data acquisition was performed using the Bio-Plex Array Reader 2200, and results were expressed as picograms per milliliter (pg/mL) based on standard curves, with data analysis conducted using Bio-Plex Manager v5.0 software. Intra- and inter-assay coefficients of variation (CVs) were consistently between 5-8%. The assay’s detection limits for each cytokine, as specified in the manufacturer’s protocol, typically fall within the pg/mL range. To ensure data integrity, several quality control (QC) measures were implemented, including validation of standard curves, dynamic range checks, and the use of both positive and negative controls to confirm the assay’s performance and specificity. Samples exhibiting outlier values were flagged for further review, and cytokine concentrations below the limit of detection (LOD) were assigned a value equal to half the LOD, minimizing potential bias. These QC steps helped maintain the reliability and accuracy of the results throughout the analysis.

### Statistical analysis

Statistical analyses were conducted using appropriate tests based on the data type. For continuous variables, such as α-diversity indices, taxonomic abundance, and cytokine levels, White’s nonparametric *t*-test, independent *t*-test, or the Mann-Whitney *U*-test were employed. Categorical variables were analyzed using Pearson’s chi-square test or Fisher’s exact test. Spearman’s rank correlation was used to assess the relationships between microbial abundances and cytokine levels, as well as inferred functions. Statistical analyses were performed using SPSS v24.0 (SPSS Inc., Chicago, IL) and STAMP v2.1.3, with graphical representations generated using R packages and GraphPad Prism v6.0. To evaluate the discriminative power of key functional taxa, random forest classification was applied, with variable importance assessed using the mean decrease Gini index. The predictive performance of the model was assessed through receiver operating characteristic (ROC) curves and area under the curve (AUC) analysis, which were conducted using the OECloud tools (https://cloud.oebiotech.com). All statistical tests were two-sided, and p-values were adjusted for multiple comparisons using the Benjamini-Hochberg method to control the False Discovery Rate (FDR). A threshold of FDR < 0.05 was considered indicative of statistical significance.

### Accession number

The sequence data from this study are deposited in the GenBank Sequence Read Archive with the accession number PRJNA1261238 (www.ncbi.nlm.nih.gov/bioproject/PRJNA1261238).

## Results

### Altered overall structure of fecal microbiota in hypertension

In this study, a total of 205 participants were recruited, consisting of 52 healthy controls and 153 individuals diagnosed with HTN. A thorough analysis of demographic and clinical characteristics revealed no statistically significant differences between the groups in terms of age, gender distribution, BMI, smoking history, alcohol consumption, medical history, blood glucose, or hypercholesterolemia (P > 0.05; **Table 1**). As anticipated, individuals in the HTN group exhibited significantly higher SBP and DBP values compared to the control group (P < 0.05), confirming the expected clinical profile of hypertension.

**Table 1:** Demographic characteristics of the participants.

Characteristic	HTN (n=153)	Con (n=52)
Age (years, Mean ± SD)	68.35 ± 4.32	69.10 ± 4.75
Gender (male/female), no.	112/41	38/14
BMI (kg/m²), Mean ± SD	25.16 ± 1.69	24.51 ± 1.86
Duration of HTN (years), Means ± SD	16.83 ± 7.93	NA
Tobacco intake, no.	25	3
Alcohol intake, no.	125	43
Antibiotics use, no.	0	0
Antihypertensive agent, no.	153	0
Gastrointestinal symptoms, no.		
Diarrhea	0	0
Constipation	15	3
Family history of HTN, no.	86	12
Blood glucose (mmol/L), Means ± SD	5.46 ± 0.92	5.71 ± 0.90
Total cholesterol (mmol/L), Means ± SD	4.96 ± 0.58	4.92 ± 0.52

BMI, body mass index; Con, Control; HTN, Hypertension; SD, standard deviation. NA, not available; no, Number.

**Figure 1:**
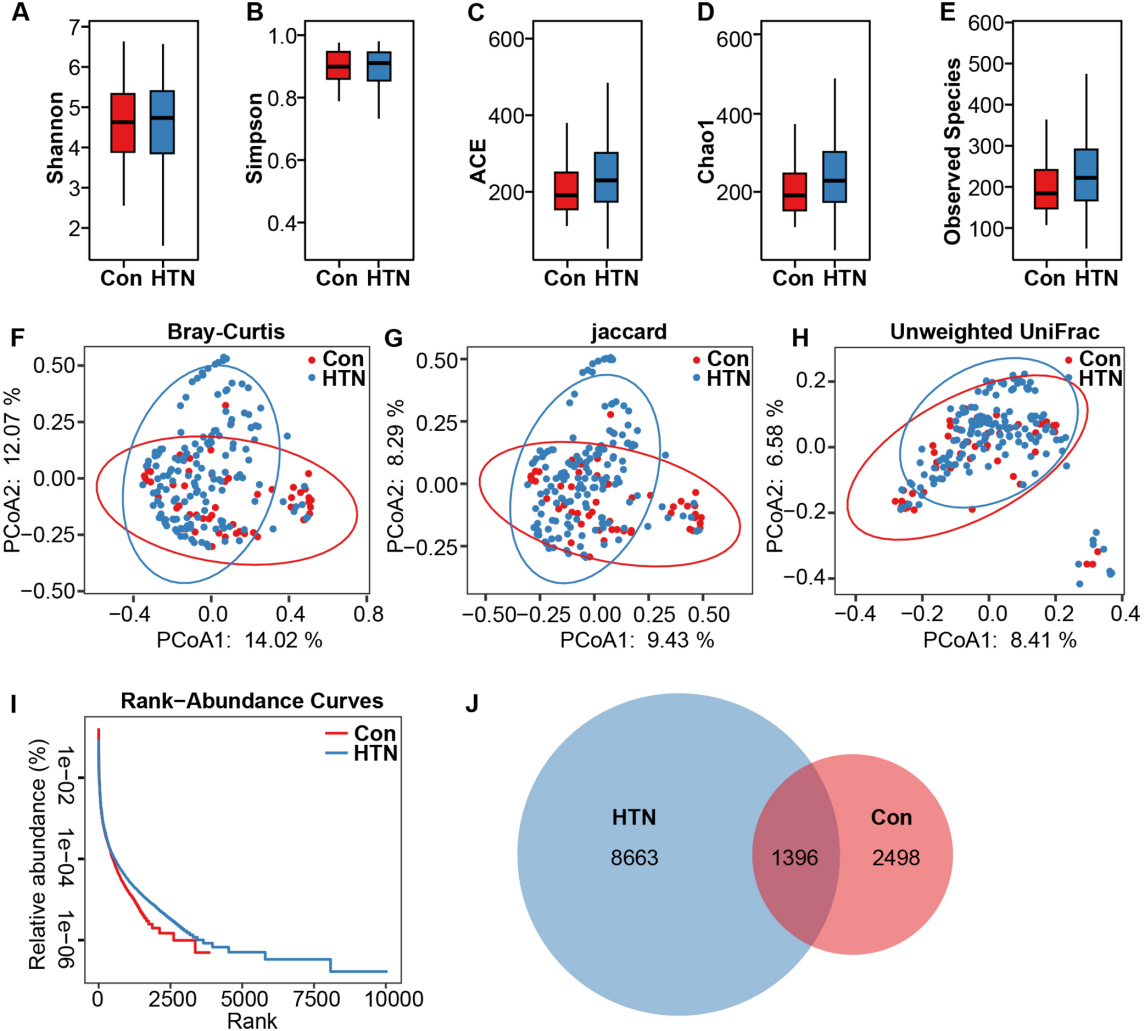
Altered overall structure of fecal microbiota in hypertension. **(A-E)** α-diversity indices (Shannon and Simpson) and richness indices (ACE, Chao1, and observed species) were employed to evaluate the overall structure of the fecal microbiota. Data are expressed as mean ± standard deviation. Inter-group comparisons were performed using unpaired two-tailed *t*-tests. **(F-H)** Principal coordinate analysis (PCoA) plots illustrate β-diversity of individual fecal microbiota based on Bray–Curtis, Jaccard, and unweighted UniFrac distances. Each symbol represents an individual sample. **(I)** Rank abundance curves display the distribution of bacterial amplicon sequence variants (ASVs) across both groups. **(J)** A Venn diagram demonstrates the overlap of ASVs between the microbiota of hypertensive patients and healthy controls.

Sequencing of fecal samples yielded 14,494,445 high-quality reads, with 3,378,923 from healthy controls and 11,115,522 from HTN patients, derived from 23,264,417 raw sequence reads, with an average of 70,705 reads per sample. For microbiota analysis, data were normalized to 38,897 reads per sample, resulting in the identification of 12,557 bacterial ASVs across the cohort. Bacterial α-diversity analyses, including Shannon, Simpson, ACE, Chao1, and observed species indices, revealed no significant differences between the HTN and healthy control groups (P > 0.05; **Figures 1A-E**). However, β-diversity analysis revealed significant differences in the fecal microbiota composition between the two groups. PCoA, based on Bray–Curtis, Jaccard, and unweighted UniFrac distances, clearly separated HTN patients from healthy controls into distinct clusters (ADONIS test: P < 0.01; **Figures 1F-H**). Rank-abundance curves indicated slightly greater richness and evenness in the HTN group compared to the control group (**Figure 1I**). Furthermore, the Venn diagram showed a greater number of unique bacterial phylotypes in the HTN group (8,663 ASVs) compared to the healthy controls (2,498 ASVs) (**Figure 1J**). These findings suggest a discernible divergence in the overall structure of the fecal microbiota between HTN patients and healthy controls.

### Altered fecal microbiota profiles in hypertension

**Figure 2:**
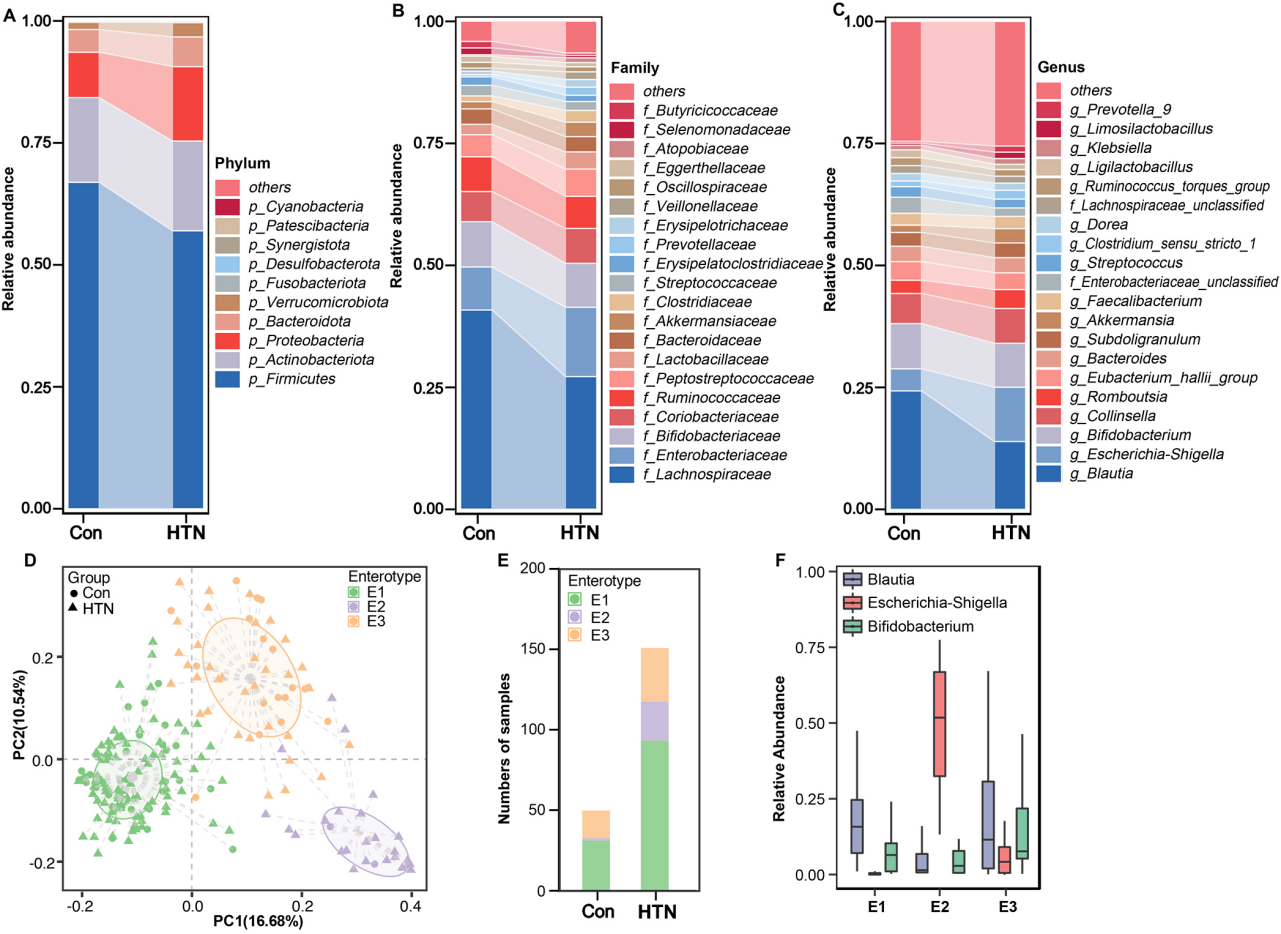
Taxonomic composition and enterotype characterization of fecal microbiota in hypertension. **(A)** Phylum-level composition. **(B)** Family-level composition. **(C)** Genus-level composition. **(D)** Principal coordinate analysis (PCoA) reveals three distinct enterotypes. Each symbol represents a sample. **(E)** Distribution of enterotypes across the hypertension and healthy control groups: hypertensive patients are predominantly associated with enterotype E2, whereas healthy controls are characterized by enterotype E1. **(F)** Relative abundance of predominant bacterial taxa within each enterotype.

After taxonomic classification of the fecal microbiota, we identified 23 phyla, 160 families, and 430 genera from both cohorts. **Figure 2** depicts the fecal microbiota landscape at the phylum (A), family (B), and genus (C) levels. The dominant phyla were Firmicutes, Actinobacteria, Proteobacteria, and Bacteroidetes, collectively accounting for over 99% of the total sequences analyzed. The fecal microbiota was further categorized into enterotypes based on genus-level composition, employing previously established methodologies (57). Three distinct enterotypes emerged: a *Blautia*-dominated enterotype (E1), an *Escherichia_Shigella*-dominated enterotype (E2), and a *Bifidobacterium*-dominated enterotype (E3) (**Figure 2D**). In the HTN group, 61.4% of samples (94/153) were classified as the E1 enterotype, 16.3% (25/153) as the E2 enterotype, and 22.3% (34/153) as the E3 enterotype. In contrast, in the healthy control group, 61.5% of samples (32/52) were classified as E1, 3.8% (2/52) as E2, and 34.7% (18/52) as E3, revealing a significant shift in microbiota composition between the two groups (**Figure 2E**). **Figure 2F** further demonstrates the relative abundance of the dominant genera within the three enterotypes, highlighting the microbiome’s variability and its potential association with HTN.

**Figure 3:**
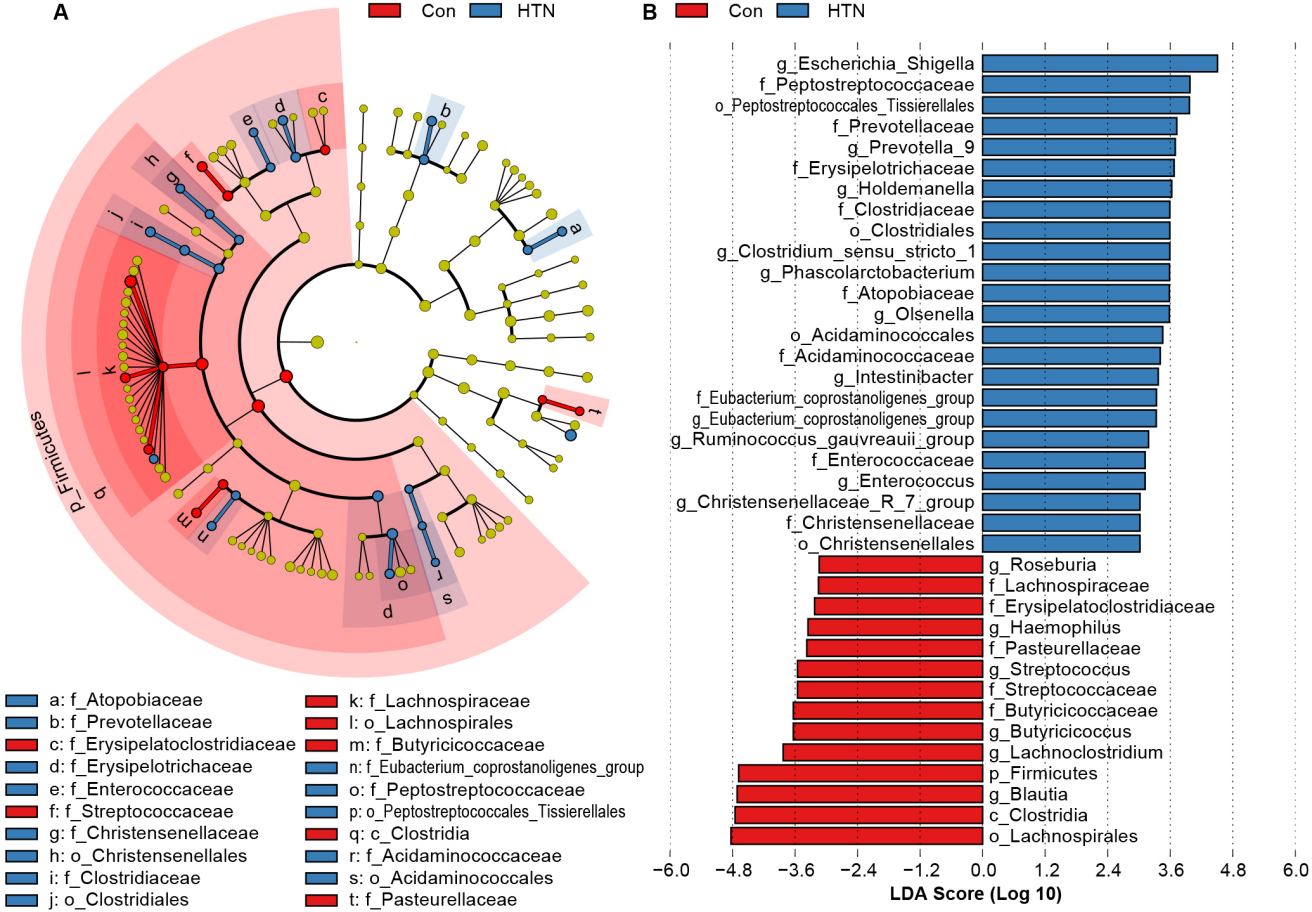
Differentially abundant bacterial taxa in the fecal microbiota of hypertensive patients vs. healthy controls. **(A)** LEfSe cladograms illustrate bacterial taxa significantly associated with either hypertensive patients (blue) or healthy controls (red). The size of each circle corresponds to the relative abundance of the bacterial taxon, with circles representing different taxonomic levels (from inner to outer: phylum, class, order, family, genus). Statistical significance was assessed using the Wilcoxon rank-sum test (P < 0.05). **(B)** Histogram displaying the distribution of Linear Discriminant Analysis (LDA) scores > 3.0 for bacterial taxa with the most significant abundance differences between hypertensive patients and healthy controls (P < 0.05).

To further elucidate the alterations in the fecal microbiota associated with HTN, we employed LEfSe to identify key bacterial taxa linked to HTN. **Figure 3** presents the results of this analysis, highlighting differentially abundant taxa that significantly distinguish HTN patients from healthy controls across various taxonomic levels (LDA score > 3, p < 0.05). The cladogram in **Figure 3A** visually summarizes the most significant compositional differences in the fecal microbiota between the two groups. Notably, genera such as *Escherichia_Shigella*, *Prevotella_9*, and *Enterococcus* were markedly more prevalent in the HTN cohort (**Figure 3B**). Conversely, butyrate-producing genera, including *Blautia*, *Butyricicoccus*, and *Roseburia*, were found to be more abundant in the healthy control group, suggesting potential protective roles against hypertension.

**Figure 4:**
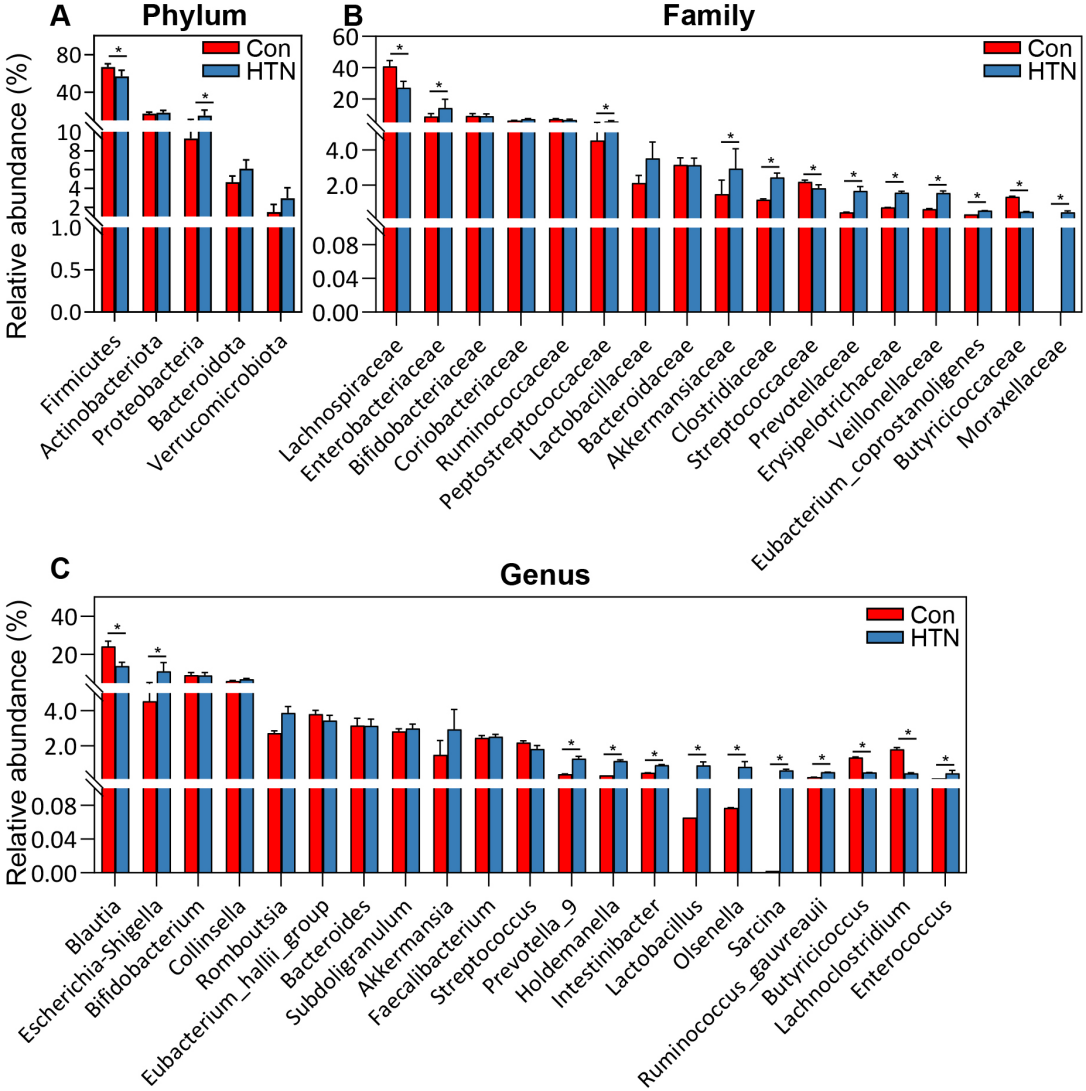
Comparative abundance of key bacterial taxa in the fecal microbiota of hypertensive patients vs. healthy controls. **(A)** Differentially abundant functional phyla. **(B)** Differentially abundant functional families. **(C)** Differentially abundant functional genera. Data are presented as mean ± standard deviation, and differences between hypertensive patients and healthy controls were analyzed using Mann-Whitney U-tests. *P < 0.05 indicates significance compared to the control group.

**Figure 5:**
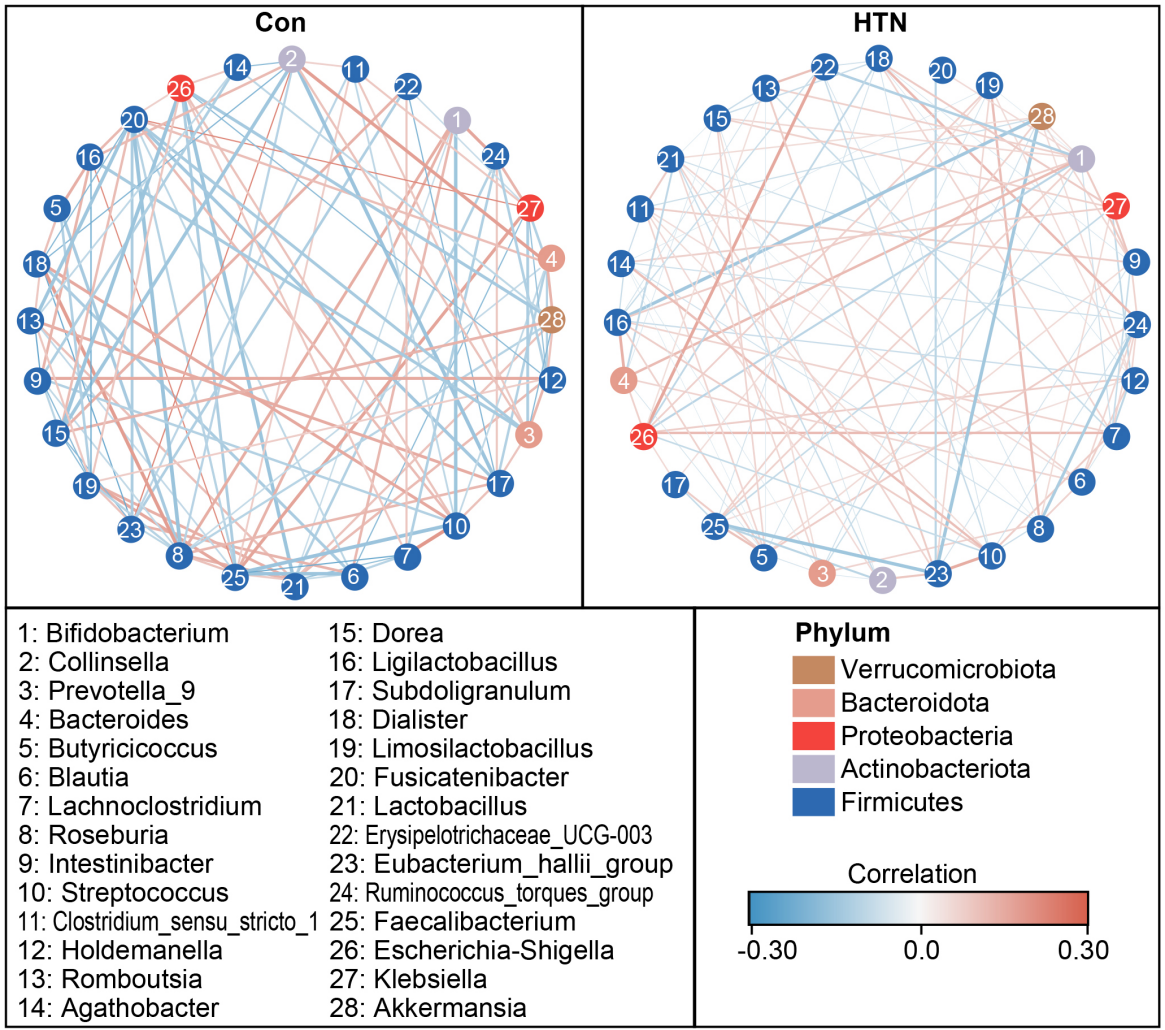
Co-occurrence network of abundant fecal genera in hypertensive patients and healthy controls. A co-occurrence network was generated using the SparCC algorithm, based on relative abundance data at the genus level, to illustrate potential ecological interactions within the microbial community. Network visualization was performed using Cytoscape version 3.6.1. Positive correlations are depicted with red lines, while negative correlations are shown with blue lines.

**Figure 4** illustrates the significant differences in fecal microbiota composition between HTN patients and healthy controls, as assessed across various taxonomic levels using MetaStats 2.0. At the phylum level, Proteobacteria were found to be significantly more abundant in HTN patients, while Firmicutes were notably less prevalent in this group compared to controls (P < 0.05; **Figure 4A**). At the family level, HTN patients exhibited increased abundances of Enterobacteriaceae, Peptostreptococcaceae, Akkermansiaceae, Clostridiaceae, Prevotellaceae, Veillonellaceae, and Moraxellaceae, while simultaneously displaying reduced levels of Lachnospiraceae, Streptococcaceae, and Butyricicoccaceae when compared to healthy controls (P < 0.05; **Figure 4B**). At the genus level, *Escherichia_Shigella*, *Prevotella_9*, *Lactobacillus*, *Ruminococcus*, and *Enterococcus* were found to be significantly elevated in HTN patients, whereas genera associated with butyrate production, such as *Blautia*, *Butyricicoccus*, and *Lachnoclostridium*, were markedly reduced (P < 0.05; **Figure 4C**). To further explore microbial interactions, we employed the SparCC algorithm to construct correlation-based networks that assess the interrelationships of microbial taxa, utilizing ASV relative abundance data across different groups (**Figure 5**). This network analysis identified 28 nodes at the genus level, representing key bacterial genera. Notably, the HTN group exhibited significantly weaker positive and negative correlations between these genera compared to the healthy control group. This finding suggests a disruption in the complex microbial interactions within the gut microbiota of individuals with hypertension. Collectively, these results underscore a pronounced dysbiosis in the fecal microbiota associated with HTN, emphasizing the perturbation of microbial balance in this condition.

**Figure 6:**
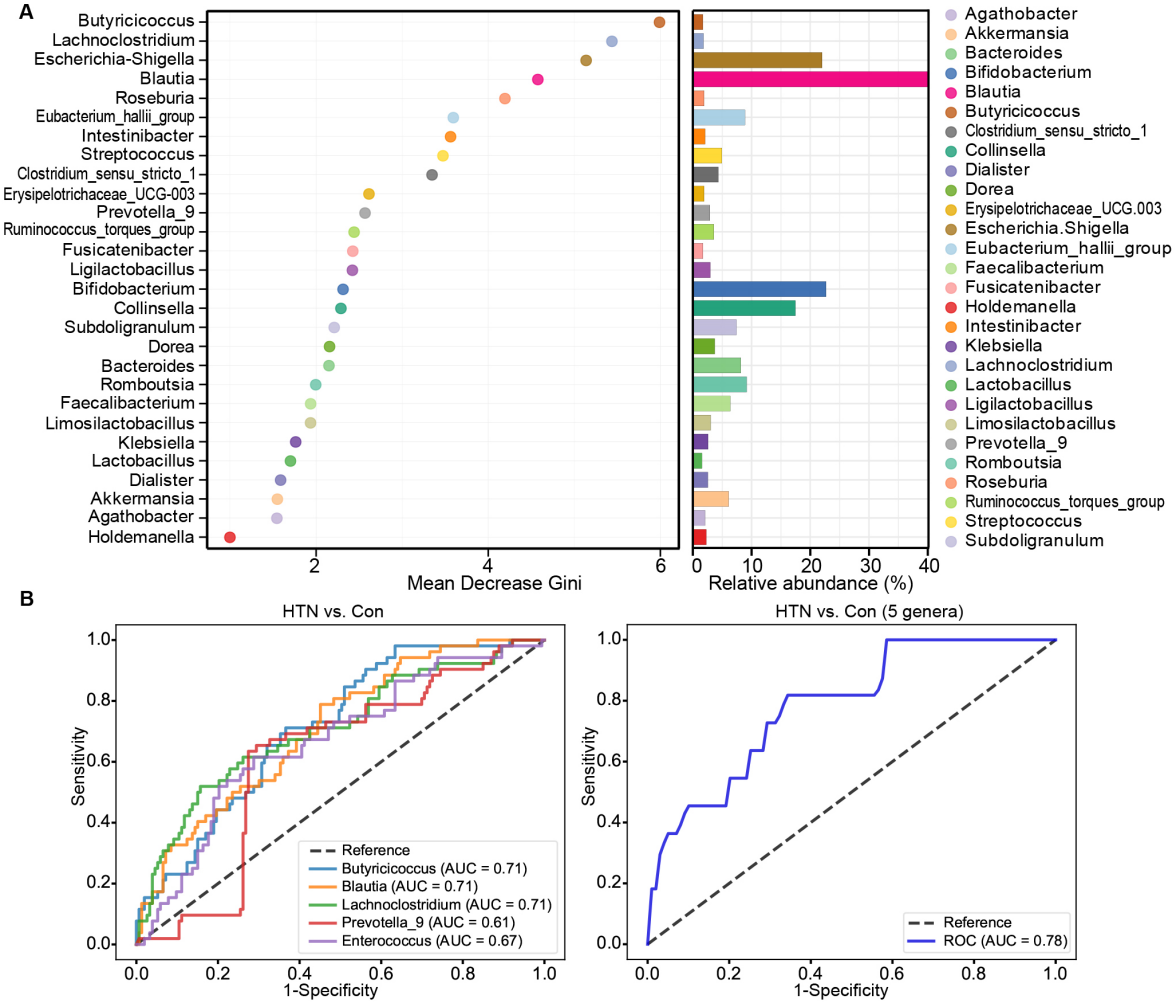
Diagnostic potential of differential genera in hypertension. **(A)** Mean Decrease Gini model for hypertension prediction, highlighting genera with greater importance in predicting hypertension. **(B)** Receiver operating characteristic (ROC) curves for differential genera, such as *Blautia*, *Butyricicoccus*, *Lachnoclostridium*, *Prevotella_9*, and *Enterococcus*, either individually or in combination, for discriminating hypertensive patients from healthy controls. AUC values represent the area under the ROC curve.

We employed Random Forest analysis to evaluate the potential of key functional bacterial taxa in distinguishing HTN patients from healthy controls. The results revealed that the Mean Decrease Gini values for *Butyricicoccus*, *Lachnoclostridium*, *Escherichia_Shigella*, *Blautia*, and *Roseburia* were notably higher, underscoring the significant contribution of these genera in predicting hypertension (**Figure 6A**). To further assess the diagnostic potential of these taxa, we conducted ROC analysis. The AUC values demonstrated the discriminatory power of each genus, with *Blautia* (AUC = 0.71), *Butyricicoccus* (AUC = 0.71), *Lachnoclostridium* (AUC = 0.71), *Prevotella_9* (AUC = 0.61), and *Enterococcus* (AUC = 0.67) showing strong accuracy in distinguishing HTN patients from controls (**Figure 6B**), while the combination of these five genera achieved even higher predictive performance (AUC = 0.78). These results suggest that these key functional bacterial genera could serve as reliable biomarkers for the early detection and non-invasive diagnosis of hypertension.

### Changed functional profiling of the fecal microbiota in hypertension

**Figure 7:**
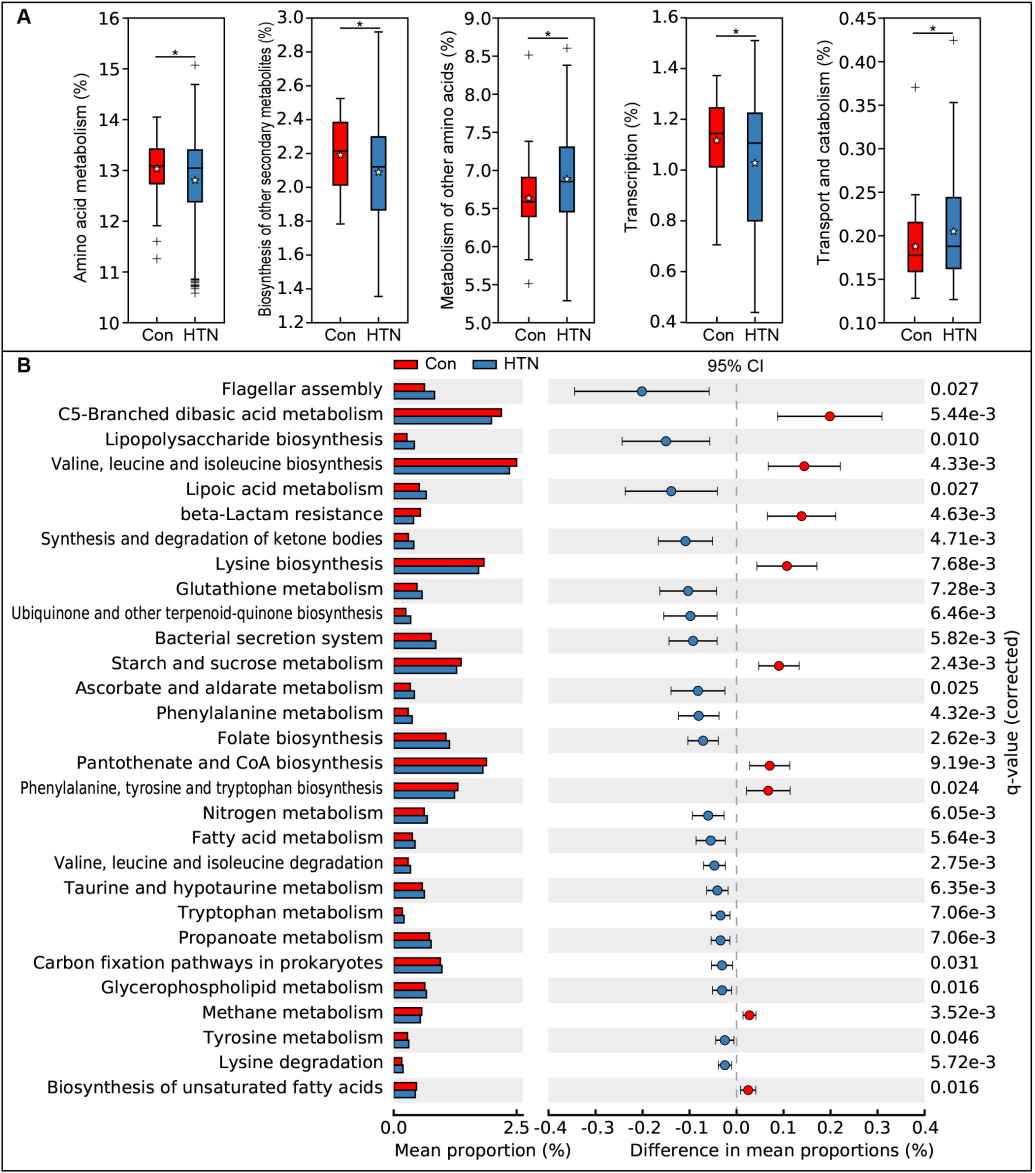
Functional profiling of fecal microbiota in hypertensive patients vs. healthy controls using PiCRUSt. Differences in bacterial functions were assessed using two-sided Welch’s t-test. Percentage comparisons of each KEGG functional category at level 2 **(A)** and level 3 **(B)** between groups are presented. Multiple testing correction was applied using the Benjamini-Hochberg method (false discovery rate, FDR) via STAMP. *P < 0.05 indicates significance compared to the control group.

We employed the PiCRUSt algorithm to investigate the functional characteristics of the microbiota associated with HTN. This tool utilizes closed-reference ASV picking to predict functional categories based on the KEGG orthology database, thereby providing insights into the metabolic and functional alterations in the fecal microbiota. The comprehensive functional profile of the microbiota linked to HTN is illustrated in **Figure 7**. Upon analyzing 64 level-2 KEGG pathways, we identified significant differences between HTN patients and healthy controls across five functional categories (P < 0.05). Two pathways, namely the metabolism of other amino acids and transport and catabolism, were enriched in the HTN group, whereas three pathways—amino acid metabolism, biosynthesis of other secondary metabolites, and transcription—were notably depleted. At the level 3 KEGG pathway classification, 29 pathways showed statistically significant variations in activity between the two groups (P < 0.05). Among these, pathways such as flagellar assembly, lipopolysaccharide biosynthesis, and lipoic acid metabolism exhibited increased activity in HTN patients. In contrast, pathways like starch and sucrose metabolism, valine, leucine, isoleucine, and lysine biosynthesis, and flagellar assembly were found to be less active in HTN group. These findings suggest that alterations in the functional potential of the fecal microbiota in HTN patients may play a pivotal role in the pathogenesis and progression of this condition.

### Correlations between differentially abundant genera and host cytokines levels

**Figure 8:**
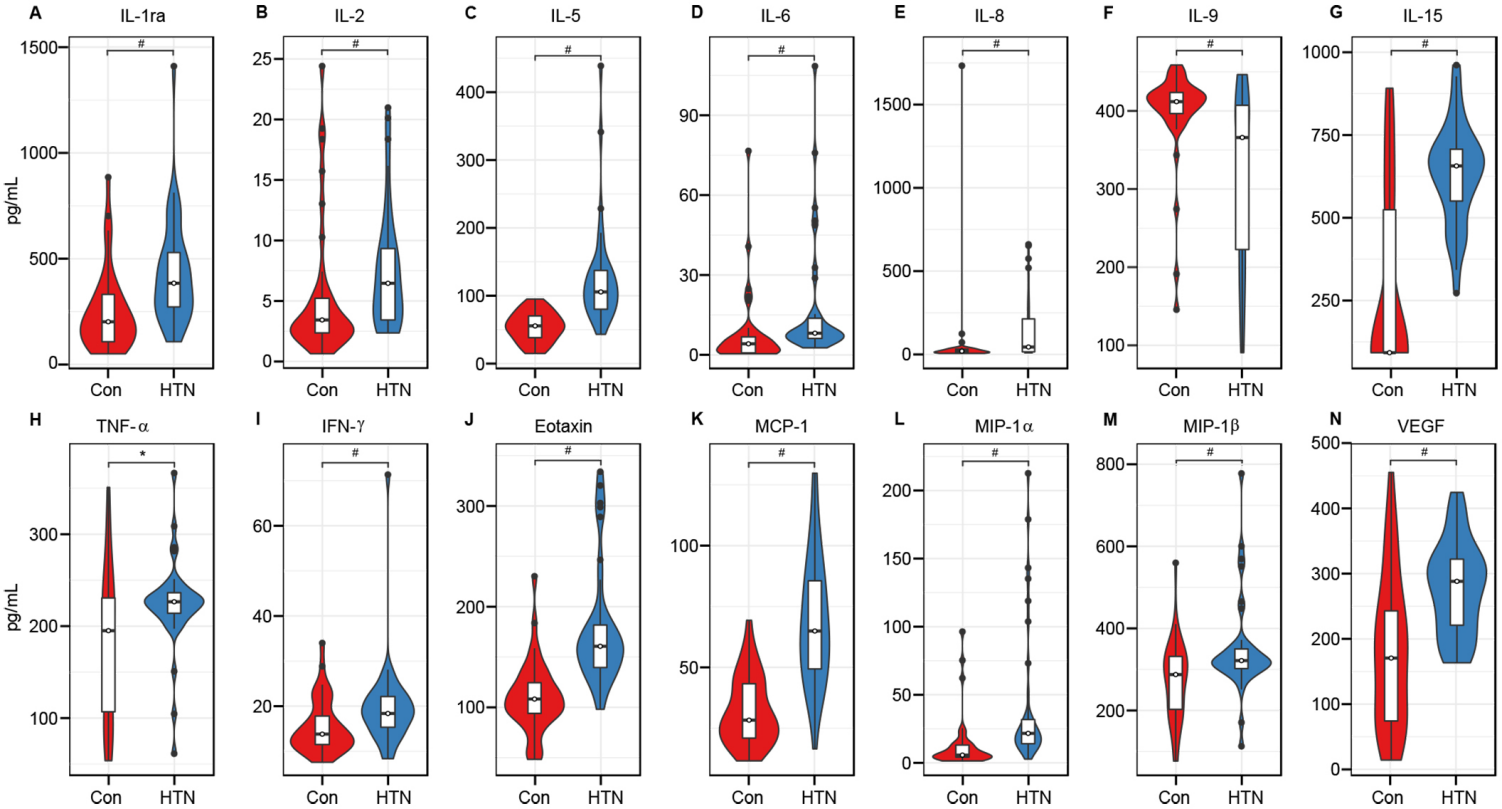
Hypertension associated immune dysfunction. The concentrations of 27 cytokines (mean ± SEM, pg/ml) were measured in hypertensive patients and healthy controls using Bio-Plex immunoassays. Results show significant increases in IL-1ra **(A)**, IL-2 **(B)**, IL-5 **(C)**, IL-6 **(D)**, IL-8 **(E)**, IL-15 **(G)**, TNF-α **(H)**, IFN-γ **(I)**, Eotaxin **(J)**, MCP-1 **(K)**, MIP-1α **(L)**, MIP-1β **(M)**, and VEGF **(N)** in hypertensive patients, while IL-9 **(F)** was significantly reduced (*P < 0.05; #P < 0.01).

**Figure 9:**
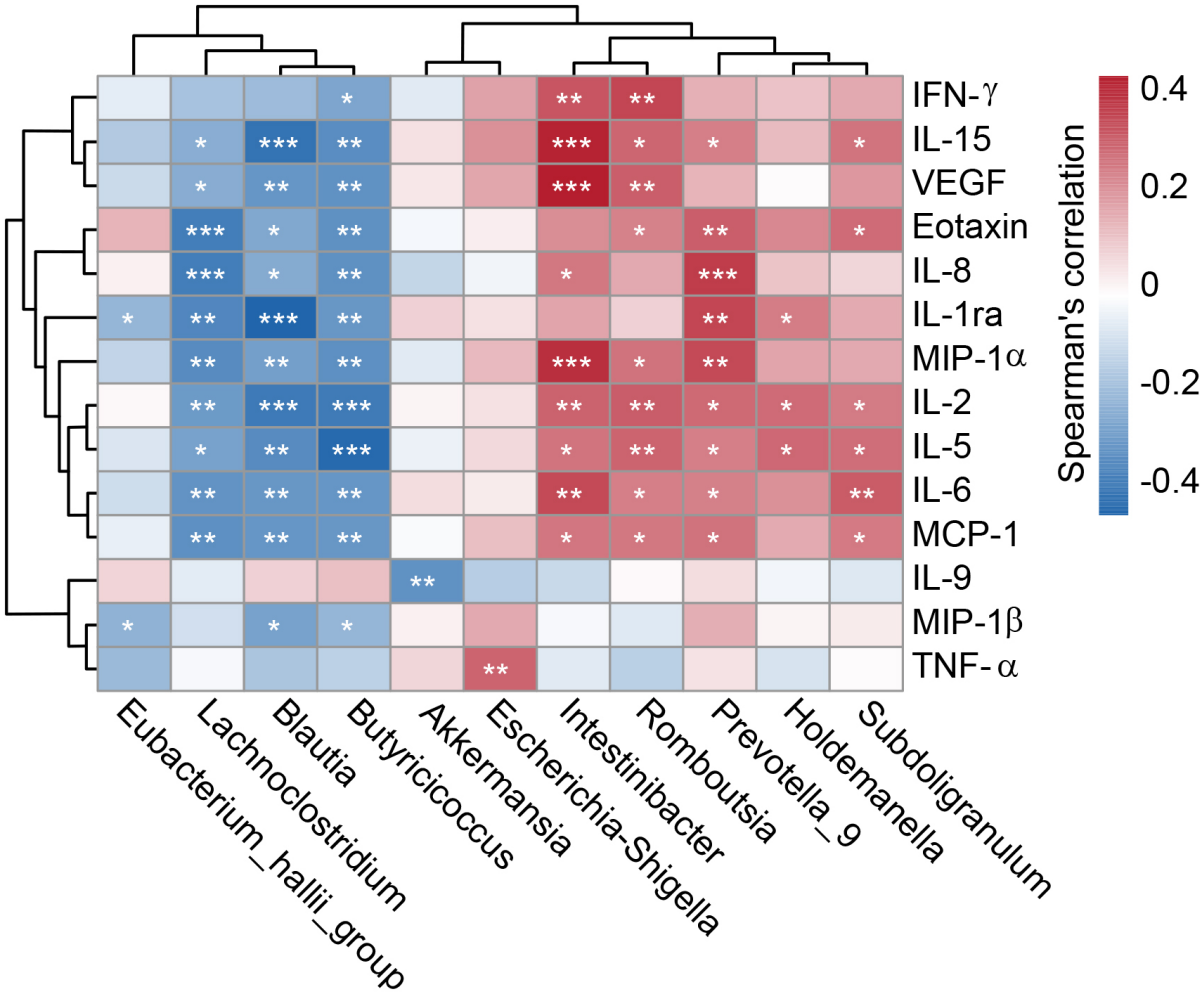
Correlation between key differential genera and immune indicators. A heatmap illustrates Spearman’s correlation coefficients between specific genera in the fecal microbiota and pro- and anti-inflammatory cytokines and chemokines in hypertensive patients. Statistical significance was assessed using Spearman’s correlation (r), with significance denoted by *P < 0.05; **P < 0.01; ***P < 0.001.

To investigate cytokine alterations between HTN patients and healthy controls, we employed the Bio-Plex Pro™ human cytokine group I panel (27-plex). As depicted in **Figure 8**, 13 cytokines, including IL-1ra, IL-2, IL-5, IL-6, IL-8, IL-15, TNF-α, IFN-γ, Eotaxin, MCP-1, MIP-1α, MIP-1β, and VEGF, were significantly upregulated in HTN patients relative to healthy controls, while IL-9 was notably downregulated (all p < 0.05). Further, we examined potential associations between host immune responses and shifts in the gut microbiota by performing Spearman’s correlation analysis (**Figure 9**). Notably, the increased abundance of opportunistic pathogenic genera in HTN patients was positively correlated with upregulated pro-inflammatory cytokines. Conversely, a reduction in butyrate-producing genera was negatively associated with these cytokines. Specifically, genera such as *Prevotella*_*9* and *Escherichia*_*Shigella*, which were elevated in HTN patients, displayed significant positive correlations with increased levels of IL-1ra, IL-8, MIP-1α, and TNF-α. In contrast, genera like *Blautia* and *Butyricicoccus*, which were diminished in HTN patients, exhibited negative correlations with pro-inflammatory cytokines, including IL-1ra, IL-2, IL-5, and IL-15. These findings highlight the role of the gut-immune axis in HTN, suggesting that the gut microbiota’s composition may contribute to chronic low-grade inflammation seen in HTN. The interactions between altered cytokine profiles and shifts in gut microbiota point to microbiota-driven mechanisms that could influence the pathophysiology of hypertension.

## Discussion

HTN as one of the leading modifiable risk factors for cardiovascular diseases, has become a pervasive global health issue, deeply affecting populations worldwide. Its prevalence continues to rise, making it a significant contributor to morbidity and mortality. What makes HTN particularly insidious is that it often develops silently, without noticeable symptoms, meaning many individuals remain unaware of their condition until severe complications, such as heart disease, stroke, or renal failure, manifest. Despite advancements in pharmacological treatments and lifestyle adjustments aimed at controlling BP, the control rates for HTN have stagnated in recent years. This is concerning, especially since many patients still struggle to achieve optimal BP control, even with the use of multiple antihypertensive agents. Therefore, there is an urgent need to develop innovative, mechanism-based approaches for identifying novel biomarkers for early HTN diagnosis and therapeutic targets to improve treatment outcomes.

One promising research avenue involves the gut microbiota, which has emerged as a critical factor in human health (58, 59). This intricate ecosystem of microorganisms plays an essential role in metabolic processes, immune regulation, and even neurological function. Gut dysbiosis has been implicated in the pathogenesis of numerous conditions, including colorectal cancer, food allergies, metabolic disorders, and central nervous system diseases (42, 45, 60–65). Recently, significant attention has been directed toward understanding the role of gut dysbiosis in the pathogenesis of HTN. Genetic models of HTN, such as Dahl salt-sensitive rats and SHRs, exhibit marked alterations in gut microbiota composition. Notably, SHRs display a nearly five-fold higher Firmicutes-to-Bacteroidetes ratio—a key indicator of dysbiosis—compared to normotensive Wistar-Kyoto (WKY) controls (66). Further supporting this link, FMT from stroke-prone SHRs (SHRSP) into antibiotic-treated WKY rats elevated SBP, whereas FMT from WKY rats to SHRSP led to a modest, albeit non-significant, reduction in BP (67). Our study presents compelling evidence linking HTN to significant gut microbiota alterations and systemic cytokine profile changes, particularly in elderly Chinese individuals. We observed distinct shifts in enterotype distribution and bacterial composition in HTN patients, characterized by an overrepresentation of opportunistic pathogens (*Escherichia_Shigella*, *Prevotella_9*, *Enterococcus*) and a depletion of beneficial, butyrate-producing genera (*Blautia*, *Butyricicoccus*, *Lachnoclostridium*). These findings align with microbial patterns seen in other metabolic disorders, such as obesity (68, 69), suggesting a shared dysbiotic mechanism in HTN pathogenesis. Prior studies have consistently reported reduced α-diversity and microbial imbalances in HTN, including increased abundance of *Prevotella*, *Escherichia_Shigella*, *Klebsiella*, *Veillonella*, and *Parabacteroides*, alongside decreased levels of SCFA-producing taxa like *Roseburia*, *Blautia*, *Eubacterium*, *Ruminococcaceae*, and *Faecalibacterium* (11, 14–16, 70–72). For instance, Li et al. used shotgun metagenomics and metabolomics to compare gut microbiota among pre-HTN, HTN, and healthy individuals, revealing reduced α-diversity in both pre-HTN and HTN groups. Notably, pre-HTN subjects exhibited a microbiota profile similar to HTN patients, with both groups predominantly classified as *Prevotella*-dominated enterotypes. HTN patients also showed significantly lower levels of SCFA-producing bacteria (*Faecalibacterium*, *Roseburia*, *Bifidobacterium*), which are critical for gut barrier integrity and anti-inflammatory signaling (15). In a large multi-ethnic cohort study utilizing machine learning models, Verhaar et al. demonstrated that the association between gut microbiota composition and BP varied significantly by ethnicity, being strongest in individuals of Dutch origin, and was further modified by age and sex, with stronger associations observed in younger individuals and women. Their analysis specifically linked lower BP to higher abundances of SCFA-producing bacteria like *Roseburia* and *Clostridium* (72). Our data corroborate these observations, demonstrating a higher prevalence of *Prevotella*_*9* and reduced abundance of *Blautia* and *Roseburia* in HTN patients. We further identified three enterotypes: *Blautia*-dominated (E1), *Escherichia_Shigella*-dominated (E2), and *Bifidobacterium*-dominated (E3). Strikingly, the E2 enterotype—enriched in pro-inflammatory *Escherichia_Shigella*—was significantly more common in HTN patients (16.3%) than controls (3.8%). This is clinically relevant, as *Escherichia_Shigella* are associated with gut barrier dysfunction and endotoxemia (73). In contrast, the E1 and E3 enterotypes, dominated by *Blautia* and *Bifidobacterium* (both linked to SCFA production and anti-inflammatory effects), were more prevalent in healthy controls. Emerging research has elucidated the critical involvement of the gut-brain axis in BP control. Mechanistically, gut dysbiosis potentiates autonomic nervous system activity through vagal signaling pathways, with specific overactivation of the sympathetic nervous system (SNS) resulting in vasoconstriction and consequent BP elevation (74). Of particular clinical relevance, high-salt diets - a well-established risk factor for HTN - induce characteristic gut microbial alterations, most notably a significant depletion of *Lactobacillus* species, whereas *L. murinus* supplementation effectively mitigates the progression of salt-sensitive HTN (22). These cumulative findings provide compelling evidence for the gut microbiota’s central role in HTN pathogenesis while highlighting potential microbiota-targeted therapeutic avenues.

Regarding the upregulated bacterial genera identified in HTN group in our study, *Prevotella* is a Gram-negative bacterium from the phylum Bacteroidetes. Previous research has suggested that an increase in *P. copri* is linked to improved glucose metabolism, potentially offering beneficial effects on metabolic health (75). For example, Chang et al. demonstrated that *P. copri*-containing consortia played a crucial role in metabolizing microbiota-directed complementary food-2 glycans, influencing weight gain and energy metabolism in gnotobiotic mice (76). *Prevotella* species are also strongly associated with chronic inflammation. Elevated levels of these bacteria have been observed in rheumatoid arthritis, HIV, bacterial vaginosis, obesity, and autism spectrum disorder (77–81), consistent with our previous findings in Chinese patients with schizophrenia comorbid with metabolic syndrome (SZ-MetS) and school-aged children with depression (63, 65). Specifically, SZ-MetS patients exhibited a significant increase in *Prevotella*_*9* abundance, which correlated with enhanced microbial LPS biosynthesis and elevated pro-inflammatory cytokines such as IL-1ra (65). Similarly, in our study of depressed children, *Prevotella* upregulation was linked to higher IL-17 levels (63), a cytokine that promotes vascular and renal inflammation in hypertension. Mechanistically, *P. copri* exacerbates intestinal inflammation by degrading the mucus layer, facilitating *Listeria monocytogenes* invasion of the epithelium, and increasing lipocalin-2 expression (82). Additionally, *Prevotella* drives Th17 pathway activation through upregulation of IL-17 and IL-23, processes that exacerbate vascular dysfunction and immune-mediated HTN in both experimental rodent models and humans (83). These dual roles of *Prevotella* in metabolism and inflammation highlight its significance in the complex interaction between gut microbiota and HTN development.

In addition to *Prevotella*, HTN patients exhibited increased abundances of *Enterococcus* and *Escherichia_Shigella*, with *Escherichia_Shigella*-dominant enterotypes being particularly prevalent. *Enterococcus* species are clinically relevant as opportunistic pathogens, frequently implicated in healthcare-associated infections. Similarly, *Escherichia_Shigella*, genetically related to *E. coli*, produces Shiga toxin and contributes to severe inflammatory conditions such as hemorrhagic colitis and gastrointestinal inflammation. Magruder et al. demonstrated a correlation between gut abundances of *Escherichia* and *Enterococcus* and subsequent bacteriuria caused by these pathogens (84). The role of *Escherichia_Shigella* in HTN is further supported by its positive association with elevated IL-1β, CXCL2, and Nod-like receptor protein 3 (NLRP3) (85, 86), reflecting its ability to infiltrate epithelial cells, induce macrophage apoptosis, and trigger IL-1β release during intestinal inflammation (87). In addition, bacteria from the Enterobacteriaceae family, including *Escherichia_Shigella*, metabolize dietary components to produce TMA, which is oxidized to TMAO in the liver. A meta-analysis of 6,176 hypertensive patients linked circulating TMAO levels to increased HTN risk (88), with mechanistic studies implicating TMAO in vascular inflammation and endothelial dysfunction (89). Notably, Jiang et al. showed that TMAO accelerates Ang II–induced vasoconstriction, exacerbating hypertensive responses (90). Beyond compositional shifts, HTN patients exhibited functional enrichment in LPS biosynthesis pathways, a mechanism central to the pro-inflammatory effects of Gram-negative bacteria like *Prevotella* and *Escherichia_Shigella*. Collectively, these findings establish a mechanistic axis linking gut dysbiosis—characterized by Gram-negative bacterial overgrowth, TMAO-mediated metabolic dysfunction, and LPS-driven inflammation—to chronic immune dysregulation and HTN pathogenesis.

In terms of the downregulated genera in HTN patients, *Blautia*, *Roseburia*, and *Butyricicoccus*, all SCFA-producing taxa, showed a reduction in abundance, consistent with previous findings (11, 14, 16, 70, 72). Wang et al. recently reported diminished *Blautia* levels in chronic kidney disease-hypertension (CKD-HTN) patients (11), while a cohort study in hemodialysis patients showed that a two-month fruit granola intervention improved BP and increased *Blautia* abundance simultaneously (91). The role of *Blautia* in metabolic health is well-established, as its baseline abundance can predict weight loss in individuals with obesity (92), and symbiotic treatments targeting this genus reduce fat mass in rodent models through SCFA-mediated pathways (93).

*Roseburia*, a key butyrate-producing bacterium, was identified as a strong predictor of both SBP and DBP in the Healthy Life in an Urban Setting (HELIUS) cohort (72), underscoring its cardiovascular importance. Similarly, *Butyricicoccus*, another butyrate-producing genus, supports colonic energy metabolism and influences BP regulation through SCFA-dependent mechanisms. SCFAs like butyrate and propionate offer multiple benefits: butyrate strengthens intestinal barriers by upregulating tight junction proteins, suppresses NF-κB-driven inflammation, and promotes Treg formation (62, 94), while propionate reduces HTN and systemic inflammation in preclinical models (95). Mechanistically, SCFAs regulate BP through G-protein coupled receptors (GPCRs) and olfactory receptor 78 (Olfr78) in vascular and renal tissues, enhancing blood vessel dilation and sodium excretion (96, 97). Butyrate also lowers BP by suppressing the renin-angiotensin system in the kidneys (98). Thus, HTN-associated depletion of SCFA-producing bacteria and reduced SCFA levels may drive disease onset and progression, positioning these microbes as potential therapeutic targets for HTN management.

HTN is increasingly recognized as a chronic inflammatory disorder, and the relationship between inflammation and HTN has become a key research area. Accumulating evidence shows that chronic low - grade inflammation drives the initiation and progression of HTN (6, 99, 100). HTN patients typically exhibited elevated systemic levels of inflammatory markers such as IL-17, IL-18, IFN-γ, TNF-α, and MCP-1 (6). Our study also found significant increases in pro-inflammatory cytokines (such as IL-1ra, IL-6) and chemokines (MCP-1) in HTN patients. Animal studies have shown that correcting imbalances in the immune microenvironment, especially in T lymphocyte subsets (101, 102), can reduce HTN and protect against vascular, cardiac, and renal damage (103). For instance, germ - free (GF) mice, which have different immune cell profiles compared to normal mice, are more prone to HTN (102, 104) and show less vascular inflammation when not exposed to certain factors like Ang II (105). This highlights the role of the microbiota in immune regulation during HTN development. Other research shows that a lack of gut microbiota worsens renal damage and inflammation (36, 106), while *Lactobacillus* supplementation can counteract the effects of a high - salt diet on BP by adjusting the gut microbiota (22). These findings reinforce the notion that HTN is intricately linked to immune dysregulation and inflammation. The gut microbiota influences HTN through dual pathways: barrier function and metabolic signaling. A dysbiotic microbiome compromises intestinal epithelial integrity, allowing LPS to translocate into the circulation and trigger chronic inflammation via TLR-mediated immune activation (107, 108). This process releases pro-inflammatory cytokines (IL-6, TNF-α) and chemokines (MCP-1), which damage vascular endothelium, promote smooth muscle cell proliferation, and impair vasoreactivity. Concurrently, microbiota-derived metabolites such as SCFAs exert anti-inflammatory effects and regulate BP by activating G-protein coupled receptors (GPR41, GPR43, GPR109A) in immune and vascular cells, enhancing sodium excretion, and suppressing the intrarenal renin-angiotensin system (109). SCFAs also inhibit histone deacetylases in immune cells, further dampening inflammatory responses. These findings establish the gut microbiota as a central regulator of immune-inflammatory pathways in HTN.

We further explored the diagnostic and therapeutic implications of key bacterial taxa. ROC analysis indicated that genera such as *Blautia*, *Butyricicoccus*, and *Enterococcus* had moderate ability to distinguish HTN patients from controls (AUC: 0.61–0.71), suggesting their potential as non-invasive biomarkers. This aligns with prior studies showing that combining microbiota composition and metabolite profiles achieves high accuracy in differentiating HTN from pre-HTN states (AUC = 0.89–0.91) (15) highlighting microbial signatures for early HTN detection. Gut microbiota-targeted interventions offer promise for HTN management. Probiotics (*Lactobacillus*, *Bifidobacterium*), prebiotics (e.g., inulin), synbiotics, dietary modifications, FMT, and microbial metabolite therapies have shown efficacy. For example, *L. plantarum* CCFM639 reduced HTN in mice (39), while *B. lactis* M8 and *L. rhamnosus* M9 modulated microbiota and metabolites to prevent BP elevation (110). A meta-analysis confirmed probiotics significantly lower SBP/DBP, particularly in patients with higher baseline BP (111). Prebiotics like inulin reduced DBP in clinical trials (112), and synbiotics lowered SBP in adults across meta-analytic datasets (113). Dietary strategies (high-fiber, DASH, Mediterranean diets) enhance microbial diversity, barrier function, and SCFA levels; a phase II trial showed prebiotic HAMSAB supplementation reduced 24-hour SBP (114). FMT also demonstrated BP-lowering effects in a clinical trial (115). Notably, drugs like losartan may act partially through microbiota modulation, restoring dysbiosis and gut integrity (116). While these findings highlight microbiota-targeted strategies as promising, further research is needed to clarify mechanisms and establish long-term safety and efficacy.

While our study offers valuable insights, several limitations must be considered. First, the cross-sectional design restricts our ability to infer causal relationships. Future longitudinal studies with larger cohorts are required to establish whether gut dysbiosis is a precursor to or a consequence of HTN. Second, the cohort included only elderly Chinese adults, limiting generalizability to other populations. Future research should enroll more diverse ethnic groups and younger individuals to assess whether the observed associations hold across demographics. Third, the lack of an independent validation cohort hinders confirmation of biomarker reliability and broader result applicability. Including such cohorts in future studies would strengthen robustness. Fourth, all hypertensive patients were on antihypertensive medications, which are known to modulate gut microbiota composition and could thus represent a potential confounding factor. Future large-scale cohort studies that include drug-naïve patients are warranted to disentangle the specific effects of hypertension from those of its treatment. Finally, although associations between gut bacteria and BP were observed, underlying mechanisms remain unclear. Mechanistic investigations—such as those using gnotobiotic animal models—are critical to establishing the causal role of specific microbes in HTN development.

## Conclusions

In summary, our study reveals that HTN is associated with distinct gut microbiota dysbiosis and systemic inflammation. This is characterized by a significant enrichment of pro-inflammatory taxa, such as *Escherichia_Shigella*, *Prevotella*_*9*, *Ruminococcus*, and *Enterococcus*, alongside a depletion of beneficial SCFAs-producing genera, including *Blautia*, *Butyricicoccus*, and *Roseburia*. These microbial imbalances strongly correlate with elevated levels of pro-inflammatory cytokines (e.g., IL-1ra, TNF-α) and chemokines (e.g., MIP-1α), suggesting a mechanistic link between gut dysbiosis and HTN development through the gut-immune axis. Notably, specific bacterial taxa, including *Blautia*, *Butyricicoccus*, *Lachnoclostridium*, *Prevotella*_*9*, and *Enterococcus*, show diagnostic potential (AUC: 0.61–0.71) as non-invasive biomarkers for HTN. The presence of a chronic low-grade inflammatory state further emphasizes the role of microbial dysbiosis in disease progression. These findings highlight the potential of microbiota-targeted approaches for HTN diagnosis and treatment, such as the development of microbial biomarkers for early detection and personalized interventions (e.g., probiotics, prebiotics, or FMT). Future research should focus on longitudinal studies to establish causality, mechanistic investigations using gnotobiotic models, and clinical trials to assess microbiota-modulating therapies, ultimately enhancing our understanding of HTN as a disorder of gut-immune crosstalk and advancing novel therapeutic strategies in HTN.

## Data Availability

The sequence data from this study are deposited in the GenBank Sequence Read Archive with the accession number PRJNA1261238 (www.ncbi.nlm.nih.gov/bioproject/PRJNA1261238).

## Supplementary material

Supplementary Figure 1 Flowchart of the study. ^#^ 17 HTN patients did not meet the inclusion criteria. ^*^ 18 healthy controls did not meet the inclusion criteria.
