# Supplementary material for: Gut microbiota dysbiosis and systemic inflammation in elderly Chinese hypertensive patients: a case-control study
Source: Front Immunol. 2025 Nov 13;16:1662578. doi: 10.3389/fimmu.2025.1662578 (PMC12657389; doi:10.3389/fimmu.2025.1662578)
Supplement: Supplementary Figure 1 — Flowchart of the study. # 17 HTN patients did not meet the inclusion criteria. * 18 healthy controls did not meet the inclusion criteria. [file DataSheet1.docx]

**Supplementary information**

Excluded (17) ^#^

Study group (153)

170 HTN patients

Excluded (18) ^*^

Blood samples

Fecal samples

Microbiota,

153 sequenced successfully

**Correlation analysis; Comparisons: α and β-diversity indices, biomarkers**

Control group (52)

70 healthy controls

Microbiota,

52 sequenced successfully

Laboratory results and other clinical indices

Blood samples

Fecal samples

Laboratory results and other clinical indices

**Figure S1** Flowchart of the study. ^#^ 17 HTN patients did not meet the inclusion criteria. ^*^ 18 healthy controls did not meet the inclusion criteria.
